# Characterization of Affitin proteolytic digestion in biorelevant media and improvement of their stabilities via protein engineering

**DOI:** 10.1038/s41598-020-76855-z

**Published:** 2020-11-12

**Authors:** Aurélie Loussouarn, Ghislaine Béhar, Frédéric Pecorari, Mikael Croyal, Axelle Renodon-Cornière

**Affiliations:** 1 CRCINA, INSERM, CNRS, Université d’Angers, Université de Nantes, Nantes, France; 2 NUN, INRA, CHU Nantes, UMR 1280, PhAN, IMAD, CRNH-O, 44000 Nantes, France; 3 CRNH-O Mass Spectrometry Core Facility, 44000 Nantes, France

**Keywords:** Protein design, Proteins, Molecular biology

## Abstract

Affitins are a novel class of small 7 kDa artificial proteins which can be used as antibody substitutes in therapeutic, diagnostic and biotechnological applications. One challenge for this type of protein agent is their behaviour in the context of oral administration. The digestive system is central, and biorelevant media have fast emerged as relevant and reliable tools for evaluating the bioavailability of drugs. This study describes, for the first time, the stability of Affitins under simulated gastric and intestinal digestion conditions. Affitins appear to be degraded into stable fragments in in vitro gastric medium. We identified cleavage sites generated by pepsin that were silenced by site-directed mutagenesis. This protein engineering allowed us to enhance Affitin properties. We showed that a mutant M1 containing a double mutation of amino acid residues 6 and 7 in H4 and C3 Affitins acquired a resistance against proteolytic digestion. In addition, these mutations were beneficial for target affinity, as well as for production yield. Finally, we found that the mutated residues kept or increased the important pH and temperature stabilities of Affitins. These improvements are particularly sought after in the development of engineered binding proteins for research tools, preclinical studies and clinical applications.

## Introduction

Proteins have become an important part of the pharmaceutical arsenal, with applications ranging from diagnosis to therapy^1–3^. However, the administration of proteins for therapeutic purposes is frequently limited to injectable routes, particularly due to their poor stability in the gastrointestinal tract. In addition to being sensitive to acidic environments related to gastrointestinal media, proteins are also degraded by the action of numerous proteolytic enzymes present in this environment.

The advantages of oral chemotherapy go beyond increased patient compliance, as flexibility of dosing schedule and cost reduction are additional benefits. However, the digestive tract does not differentiate between therapeutic and non-therapeutic proteins, which are both digested. Thus, the oral delivery of therapeutic protein drugs has always been a challenge and many efforts have been made to increase the proteolytic stability of these therapeutic agents^4,5^. One of the approaches is rational protein engineering in which defined mutations are inserted based on protein structure information and biochemical knowledge.

In that respect, elaborating in vitro biorelevant media that simulate human digestion has become increasingly important in nutritional and pharmaceutical research and development^6–9^. Such media should facilitate the prediction of food and drug behaviours in terms of solubility and stability, minimizing the need for animal and human testing.

Small (4–12 kDa) recombinant binder proteins have the potential to become a new class of therapeutic agents that bridge the gap between monoclonal antibodies and small molecule drugs^1,10–13^. Their clinical evaluations as antibody mimetics are ongoing. They have many advantages, such as selectivity, stability, and low production costs, whether from bacterial or chemical production. Of these binders, Affitins^14^, which are derived from the hyperthermostable Sac7d protein hosted by the archaeon *Sulfolobus acidocaldarius*, have been found of interest for therapeutic purposes^15–17^ but also for biotechnological applications such as detection reagents^17–20^ and affinity chromatography^21^. In previous works, a thermostable Affitin H4 (H4) directed against hen egg white lysozyme was identified^22^ and used for different studies as proof of concept^18,19,23^.

To evaluate the behaviour of Affitins in the context of oral administration, here we investigate their stability by using simulated gastric and intestinal physiological in vitro conditions. Using these media, we observed stable digested fragments of H4 in the gastric environment. After identifying the cleavage sites by mass spectrometry-based approaches, site-directed mutagenesis was performed to prevent proteolytic degradation. Subsequently, the recombinant mutant proteins were purified, and their biochemical properties were characterized and compared with wild-type proteins.

Our study highlights that simulated gastrointestinal media may predict the oral digestion of therapeutic proteins. It also provides evidence that protein engineering at the identified cleavage sites, whose common strategy is site-directed mutagenesis, is an important tool for changing protein properties and improving both proteolytic stability and their efficacy, thermal stability and/or production level.

## Results

### Gastric and intestinal digestion of H4

Affitin H4 (0.5 mg/mL) was incubated at 37 °C for 30 min in simulated in vitro gastric (FaSSGF) and intestinal (FaSSIF) fluids containing the corresponding enzymes to evaluate its proteolytic degradation. Pepsin is the only proteolytic enzyme in the human stomach. Its concentration fluctuates between individuals and digestion states, and ranges from 0.025 to 3.2 mg/mL^24–26^. Commercial Pancreatin mixture contains all the key enzymes (protease, amylase and lipase) found in the intestinal lumen and is frequently used in simulated intestinal media at concentrations ranging from 0.01 to 10 mg/mL^24–26^. The extent of H4 enzymatic digestions was estimated by SDS-PAGE under reducing conditions. The control samples without digestion were obtained by inactivating digestive enzymatic activity before including Affitins, i.e. adding Na_2_CO_3_ for gastric digestions and heating the samples for 10 min at 100 °C for intestinal digestions.

**Figure 1:**
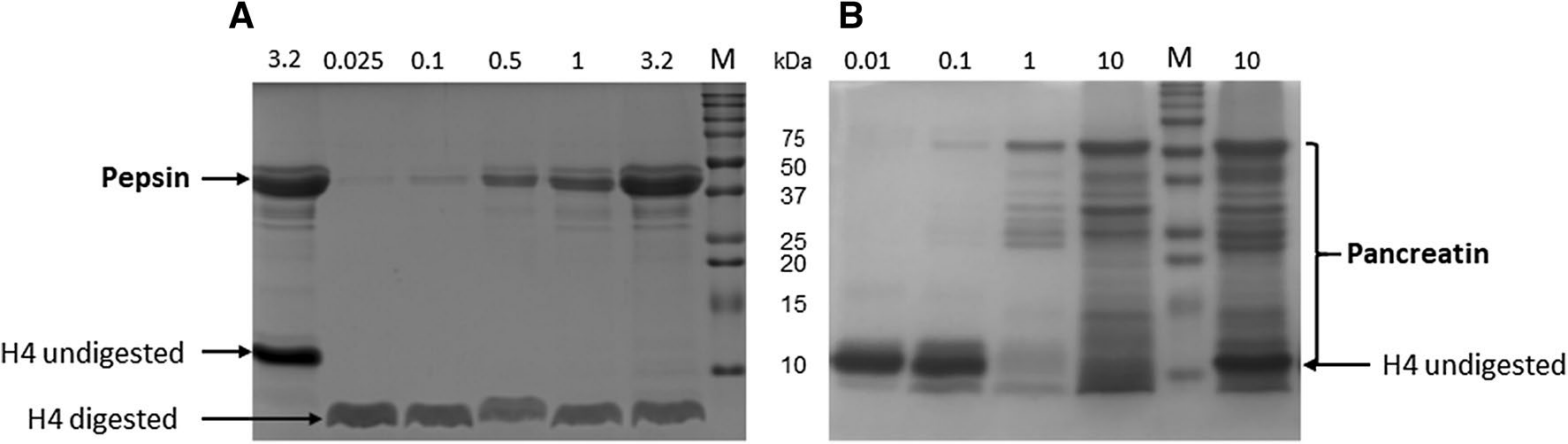
Proteolytic digestion of H4 by (**A**) pepsin in FaSSGF or (**B**) pancreatin in FaSSIF. Proteins (0.5 mg/mL, 5 µg) were separated by SDS − PAGE under reducing conditions and stained with Coomassie brilliant blue after they were incubated for 30 min (**A**) with different pepsin concentrations in FaSSGF medium (pH 1.6) [lane 1: control without H4 digestion (3.2 mg/mL denatured pepsin); lane 2: 0.025 mg/mL pepsin; lane 3: 0.1 mg/mL; lane 4: 0.5 mg/mL; lane 5: 1 mg/mL; lane 6: 3.2 mg/mL; lane 7: Molecular mass markers (M)] or (**B**) with different pancreatin concentrations in FaSSIF medium (pH 6.5) [lane 1: 0.01 mg/mL pancreatin; lane 2: 0.1 mg/mL; lane 3: 1 mg/mL; lane 4: 10 mg/mL; lane 5: molecular mass markers (M); lane 6: control without H4 digestion (10 mg/mL denatured pancreatin)]. Full-length gels are shown in supplementary Fig. S1.

Whatever protease concentration used, H4 was degraded into a single lower molecular weight band in the gastric medium (Fig. 1A). We further observed that the remaining fragment was stable and completely resistant to proteolysis by pepsin throughout digestion in the stomach and for more than 24 h. In contrast, H4 was fully digested and disappeared in the intestinal medium (Fig. 1B).

Given the total H4 degradation in simulated intestinal fluid, it was not possible to consider H4 engineering to improve its proteolytic degradation. On the other hand, the apparent stability of H4 in simulated gastric fluid encouraged us to investigate additional rational protein designs.

### Identifying the cleavage sites after pepsin digestion of H4

**Figure 2:**
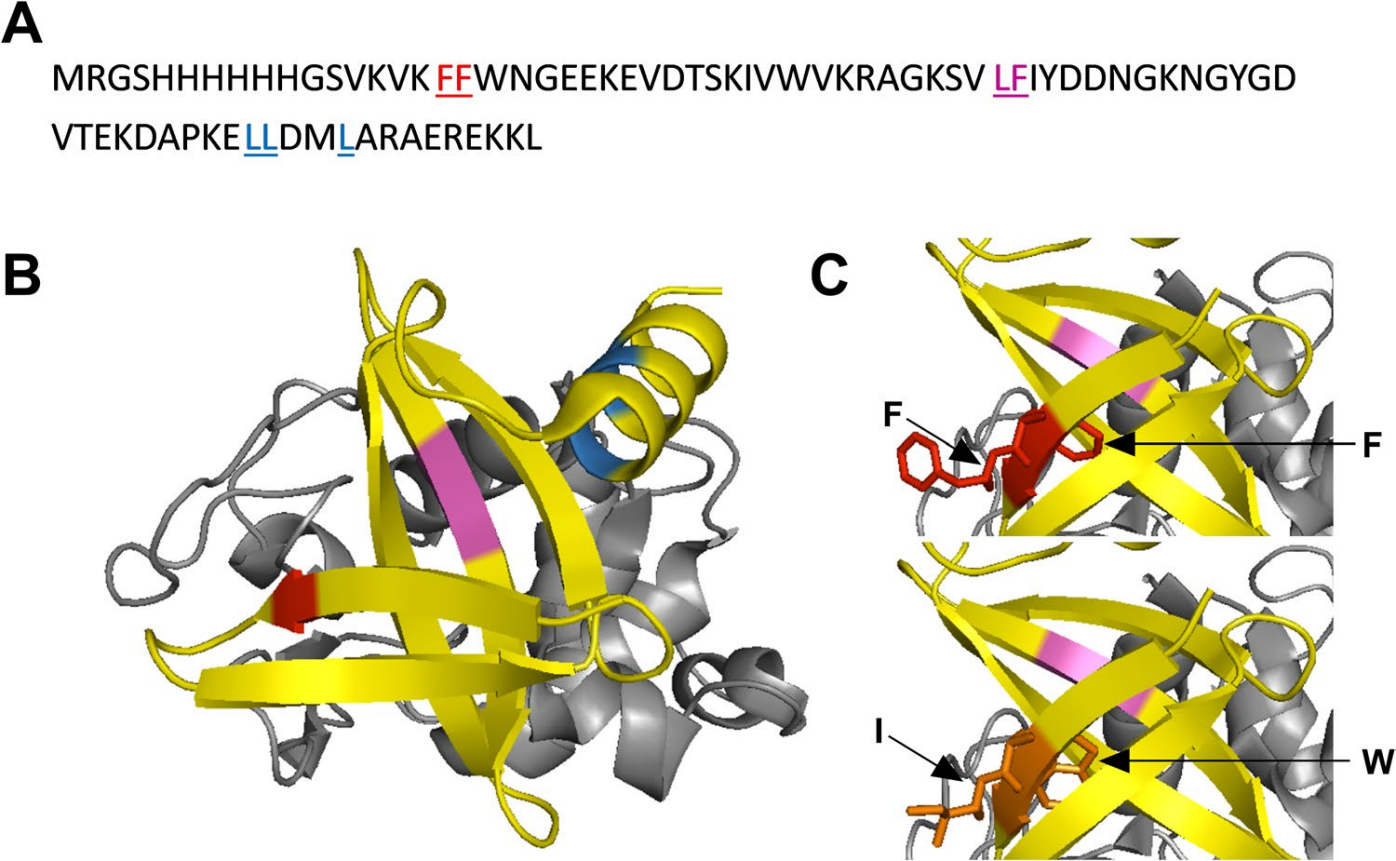
Location of amino acids involved in pepsin digestion of H4 at pH 1.3. The positions of aromatic residues implicated in the pepsin digestion of H4 are shown in the 3-D structure of the central domain determined by X-ray crystallography (PDB: 4CJ2)^23^. (**A**) Amino acid sequence of H4 with in silico predicted digestion sites underlined. (**B**) The amino acids F6 and F7 (red) are on the β-sheet. The amino acids L31 and F32 (magenta) are located on the binding interface with the lysozyme (grey). The amino acids L54, L55 and L58 (blue) are on the C-terminal α-helix. (**C**) The amino acids F6 and F7 (red) in H4 were substituted by a Tryptophan (W) and an Isoleucine (I) respectively (orange) in mutant M1. All the structures were drawn using PyMOL software (the PyMOL Molecular Graphics System, version 2.3.2, Schrödinger, LLC, https://www.pymol.org/).

To identify the cleavage sites in the H4 sequence, two complementary approaches were used. In silico digestion was first performed using the ExpasyMass software (https://web.expasy.org/peptide_mass/) which theoretically predicted the residues cleaved after pepsin proteolysis at pH 1.3. Three cleavage sites were found, as indicated in Fig. 2A,B. The first occurred on the first β sheet between phenylalanine 6 (F6) and F7 residues (marked in red). The second occurred between leucine 31 (L31) and F32 on the central β sheet, known to be involved in the target interaction (marked in green). The last site was situated in the C-terminal α helix and implicated residues L54, L55 and L58 (marked in blue).

**Figure 3:**
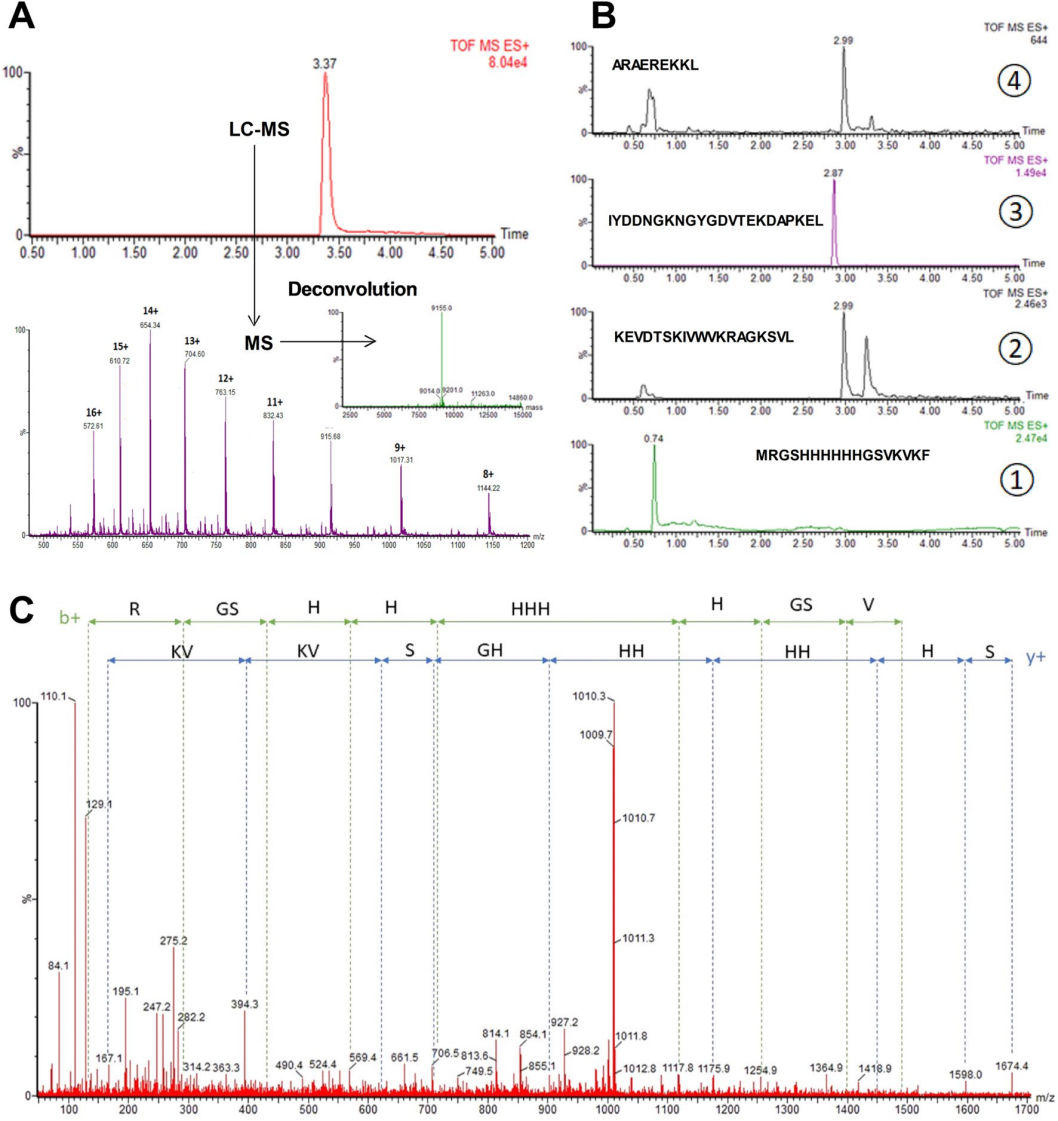
Mass spectrometry analysis of undigested and digested H4. (**A**) LC–MS chromatogram obtained for undigested H4 and underlying mass spectrum with deconvoluted mass spectra. (**B**) Extracted ion chromatograms obtained after H4 pepsin proteolysis. (**C**) MS/MS spectrum of the first peptide fragment of digested H4 (MRGSHHHHHHGSVKVKF, MS/MS of the doubly charged precursor at *m*/*z* 678.8).

Mass spectrometry experiments were then performed to validate our in silico observations and identify the digested fragments produced after H4 pepsin proteolysis. As shown in Fig. 3A, undigested H4 analysis showed only one chromatographic peak at 3.37 min. Mass spectrum deconvolution revealed the presence of a single molecule with a molecular weight of 9155 Da, corresponding to H4 and indicating that pepsin activity was efficiently stopped. In contrast, digested H4 analysis indicated the existence of 4 major fragments with specific retention times, and confirming the presence of the three cleavage sites predicted in silico (Fig. 3B). Major precursor ions related to peptide fragments were then selected for MS/MS fragmentations (Fig. 3C) and the fragmentation patterns obtained after MS/MS analyses clearly confirmed the peptide sequences expected.

### Protein engineering of H4 by site-directed mutagenesis

To improve the proteolytic stability of Affitins, rational protein engineering by site-directed mutagenesis was implemented in this study. Different criteria were considered to select the mutated amino acids. First, based on protein structure and function knowledge^27^, we selected residue substitutions known to conserve the wild-type properties for either conformation and activity. Second, we took into consideration the susceptibility of two specific residues to be cleaved by pepsin^28,29^. Three mutants M1, M2 and M3 (Table 1 and supplementary Table S1) were thus designed to investigate the impact of the mutated amino acids on proteolytic degradation by pepsin. M1 corresponded to the double mutations of F6 to tryptophan (W6) and F7 to isoleucine (I7), M2 carried the double mutations L31 to I31 and F32 to W32, and M3 had three mutations: L54 to I54, L55 to valine (V55) and L58 to I58. Directed mutagenesis was performed by PCR with custom-synthesized primers (supplementary Table S2). The mutants were constructed from H4 plasmid^22^. After cloning and transformation into bacteria, the mutations were confirmed by DNA sequencing. The proteins were then overexpressed in an *E. coli* host and purified by affinity chromatography (Ni–NTA) followed by size-exclusion chromatography. The Affitins were more than 95% pure based on SDS-PAGE analysis (supplementary Fig. S2). Interestingly, compared with the wild-type counterpart (Table 1), the production yield of M1 was increased more than fourfold from 10 mg to 44.5 mg per litre of culture, while the production yields for M2 and M3 were slightly reduced or similar (respectively 5.5 and 8.3 mg).

**Table 1:** Characteristics of wild-type and mutated H4.

	Mutations	MM^a^	EC^b^	Yield^c^	K_D_^d^ [Chi2^e^]
H4	/	9146.34	13,980	10.0 ± 0.1	6.37 [5.16]
M1	F6W-F7I	9151.36	19,480	44.5 ± 2.0	3.98 [2.57]
M2	L31I-F32W	9185.37	19,480	5.5 ± 0.2	440 [0.10]
M3	L54I-L55V-L58I	9132.31	13,980	8.3 ± 1.4	7.5 [2.51]

^a^Molecular mass (MM) in g mol^−1^.

^b^Molar extinction coefficient (EC) at 280 nm in M^−1^ cm^−1^.

^c^mg of Affitin purified from 1 L of growth medium.

^d^Equilibrium dissociation constant in nM.

^e^Chi2 in RU measured by SPR measurements with concentrations of Affitin from 15.6 to 500 nM.

### Gastric digestion of H4 mutants

**Figure 4:**
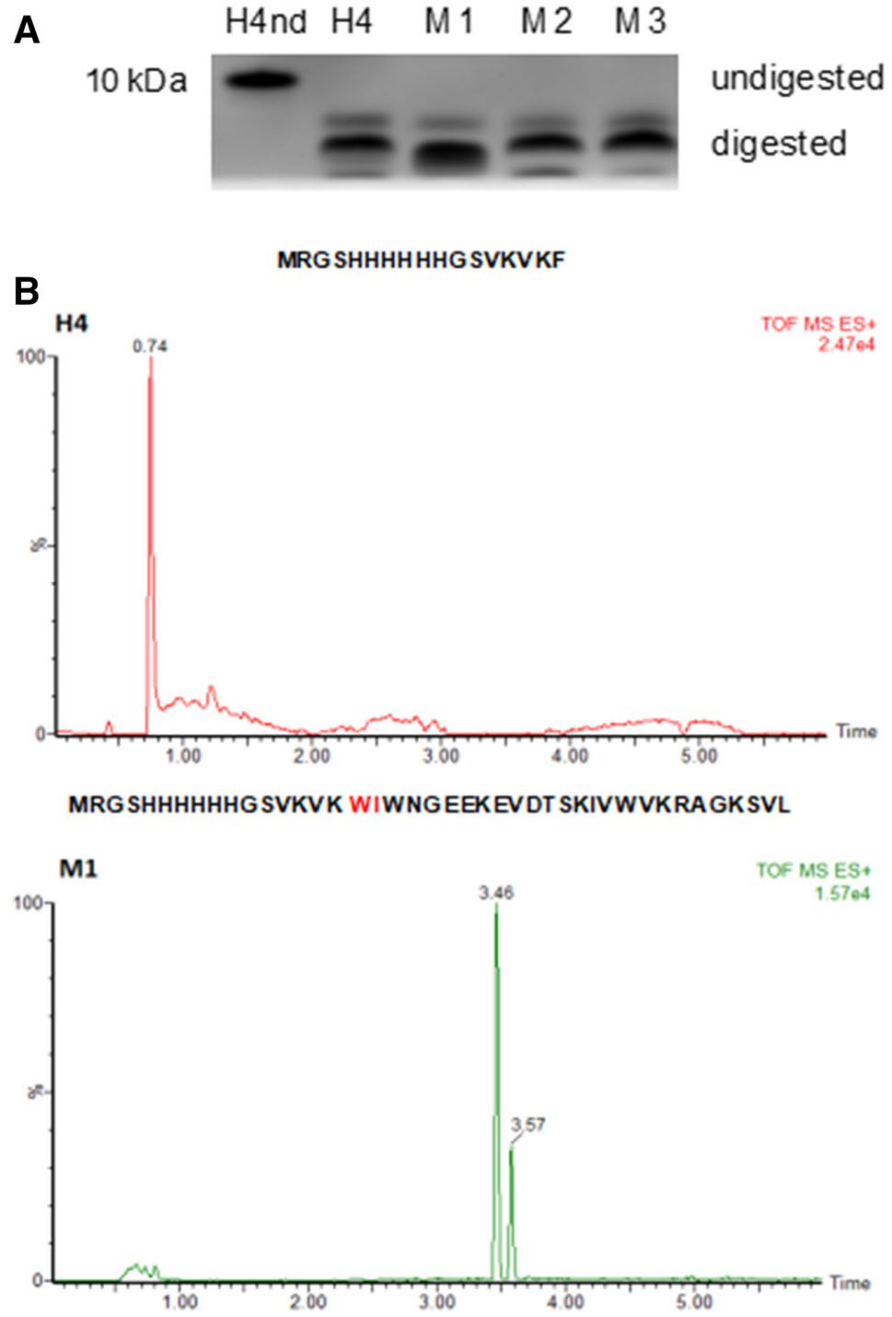
Analysis of the pepsin digestion of (**A**) H4 and mutants by tricine SDS-PAGE and (**B**) H4 and M1 by mass spectrometry. (**A**) Tricine-SDS-PAGE analysis of wild-type and mutated H4 Affitins (0.5 mg/mL, 5 µg) incubated for 30 min in presence of pepsin (0.1 mg/mL). M: Molecular mass markers; lane 1: H4 without digestion (nd); lane 2: H4; lane 3: M1; lane 4: M2; lane 5: M3. Full-length gel is shown in supplementary information (Fig. S3). (**B**) Extracted ion chromatograms corresponding to the first peptide generated with pepsin for H4 and M1.

First, we verified whether the inserted mutations conferred resistance to gastric digestion on the mutants. As only one of the three cleavage sites identified was modified in each mutant, we did not expect to totally protect the proteins from digestion. To better analyse the resulting fragments produced after pepsin incubation of the Affitins, more resolving SDS-PAGE experiments were performed using tricine instead of glycine SDS-PAGE gels. The presence of both glycerol and urea also resulted in better separation of each fragment. The separation of the fragments produced by pepsin digestion of H4 and the three mutants M1, M2 and M3, monitored by tricine SDS–PAGE gels, is shown in Fig. 4A. They all presented three bands. Even though the tricine SDS–PAGE analysis made it possible to differentiate the different fragments, it was still not sufficiently resolved to allow us to associate one band with each fragment. Due to low molecular weight, some fragments appeared near the dye front. Nevertheless, we noticed that the digestion pattern of M1 (lane 3) was different from that of H4 (lane 2) while those of M2 (lane 4) and M3 (lane 5) seemed similar. It should be noted that the main band of M1 after digestion migrated faster, while the resulting fragment after digestion was of a larger size. This result can be explained by the presence of urea, which modified the migration of the fragments. In the absence of urea, the main band of M1 after digestion migrated less quickly through the gel than that of H4, suggesting a larger fragment size, as expected (supplementary Fig. S4).

Mass spectrometry experiments confirmed that the mutations introduced in M2 and M3 did not protect them against pepsin proteolysis. Interestingly, mass spectrometry spectra showed that changing the residues 6 and 7 from F-F to W-I in M1 conferred resistance to pepsin cleavage (Fig. 4B). When a manual peptide search was carried out, the peptide MRGSHHHHHHGSVKVKW was not found, whereas the peptide MRGSHHHHHHGSVKVKWIWNGEEKEVDTSKIVWVKRAGKSVL appeared at 3.46 min (Fig. 4B). Of note, MRGSHHHHHHGSVKVKWIWNGEEKEVDTSKIVWVKRAGKSVL generated two chromatographic peaks (3.46 min and 3.57 min) with similar m/z, which could stem from isomeric forms.

### Effect of mutations on protein binding

**Figure 5:**
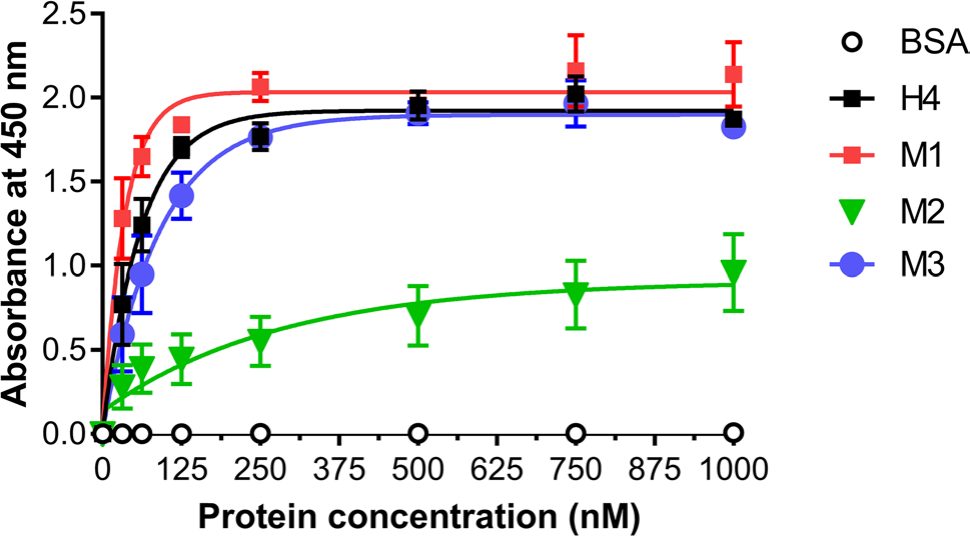
Specific binding of H4 and mutants to lysozyme using ELISA. After lysozyme immobilization on plates, increasing concentrations of Affitins from 31.25 to 1000 nM were incubated for 1 h at room temperature and their binding was detected with HRP-conjugated anti-RGS-6His antibody and absorbance at 450 nm. Commercial BSA were used as negative controls. Results are presented as means and error bars represent standard deviations of absorbances achieved from at least three experiments.

To assess whether the mutations introduced interfered with the Affitin’s binding properties, enzyme-linked immunosorbent assay (ELISA) experiments were carried out. We investigated the interaction of H4 and its mutants with its specific target lysozyme. Figure 5 revealed that the M2 mutant exhibited a strong decrease in lysozyme binding compared with H4 and M3. In contrast, the binding efficiency of M1 appeared to be increased.

As an alternative approach to evaluating the interaction of Affitins with lysozyme, Surface Plasmon Resonance (SPR) experiments were monitored at 25 °C. Lysozyme was immobilized on a CM5 chip by amine coupling (200 RU) and the association (2 min) and dissociation (7 min) of each Affitin were measured. As shown in Table 1, and compared with the equilibrium dissociation constant K_D_ of H4 (6.37 nM), M1 displayed the highest apparent affinity towards lysozyme (K_D_ of 3.98 nM), in particular through a twofold increase in its apparent association rate constant. No tangible changes in kinetic values of M3 were observed compared with the wild-type, while M2 displayed a dramatically decreased apparent affinity (K_D_ of 440 nM).

### Effect of mutations on the secondary structure of proteins and pH stability

**Figure 6:**
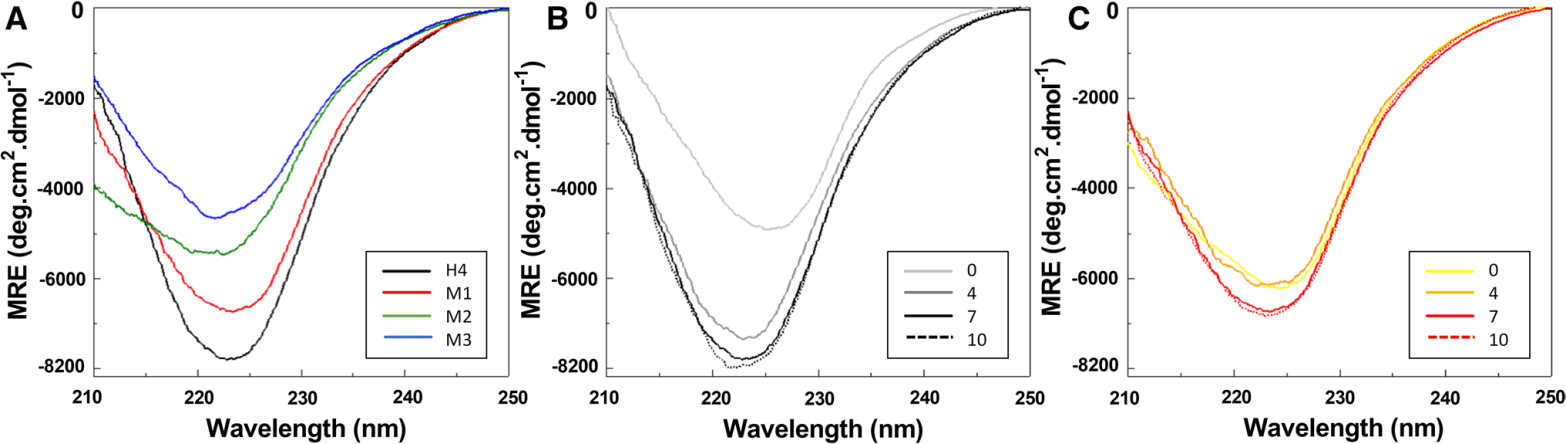
Secondary structure of H4 and mutants by circular dichroism. Far-UV CD spectra of 0.25 mg/mL H4 (wild-type or mutants) were recorded at 20 °C in 20 mM sodium phosphate buffer at pH 7 (**A**) or at different pH ranging from 0 to 10 for H4 (**B**) and M1 (**C**).

To inspect whether the secondary structure of Affitins was changed by the mutations, far-UV CD spectra of the wild-type and mutated proteins were recorded from 210 to 250 nm at 20 °C in 20 mM sodium phosphate buffer, pH 7. As previously described^30–32^, H4 displayed a spectrum characteristic of β-stranded proteins with an α helix contribution, which has a large negative band with a minimum near 222 nm (Fig. 6A). The far-UV CD spectra of mutants showed that the mutations did not seem to disrupt the secondary structures of the proteins (Fig. 6A). These results suggest that the overall secondary structure of the mutants was not changed significantly by the mutations. Nevertheless, whereas the mutants still adopted a well-defined fold, the resulting conformations appeared to be different from that of the wild-type H4.

pH stability was also assessed by incubating the proteins overnight in 20 mM sodium phosphate buffer at pH ranging from 0 to 10. CD spectra indicated that the secondary structure of mutants (Fig. 6C and supplementary Fig. S5) remained largely stable, as with H4 (Fig. 6B). It is noteworthy that M1 even seemed to display greater pH stability at pH 0 (Fig. 6C) than H4 (Fig. 6B).

### Effect of mutations on thermostability

Thermostability was evaluated by two methods. First, CD measurements at 222 nm in potassium phosphate buffer, pH 7.4 were recorded at temperatures ranging from 20 to 95 °C. Second, the binding properties of Affitins were investigated using ELISA after they were pre-incubated for 1 h at temperatures ranging from room temperature to 90 °C prior to their incubation with lysozyme.

**Figure 7:**
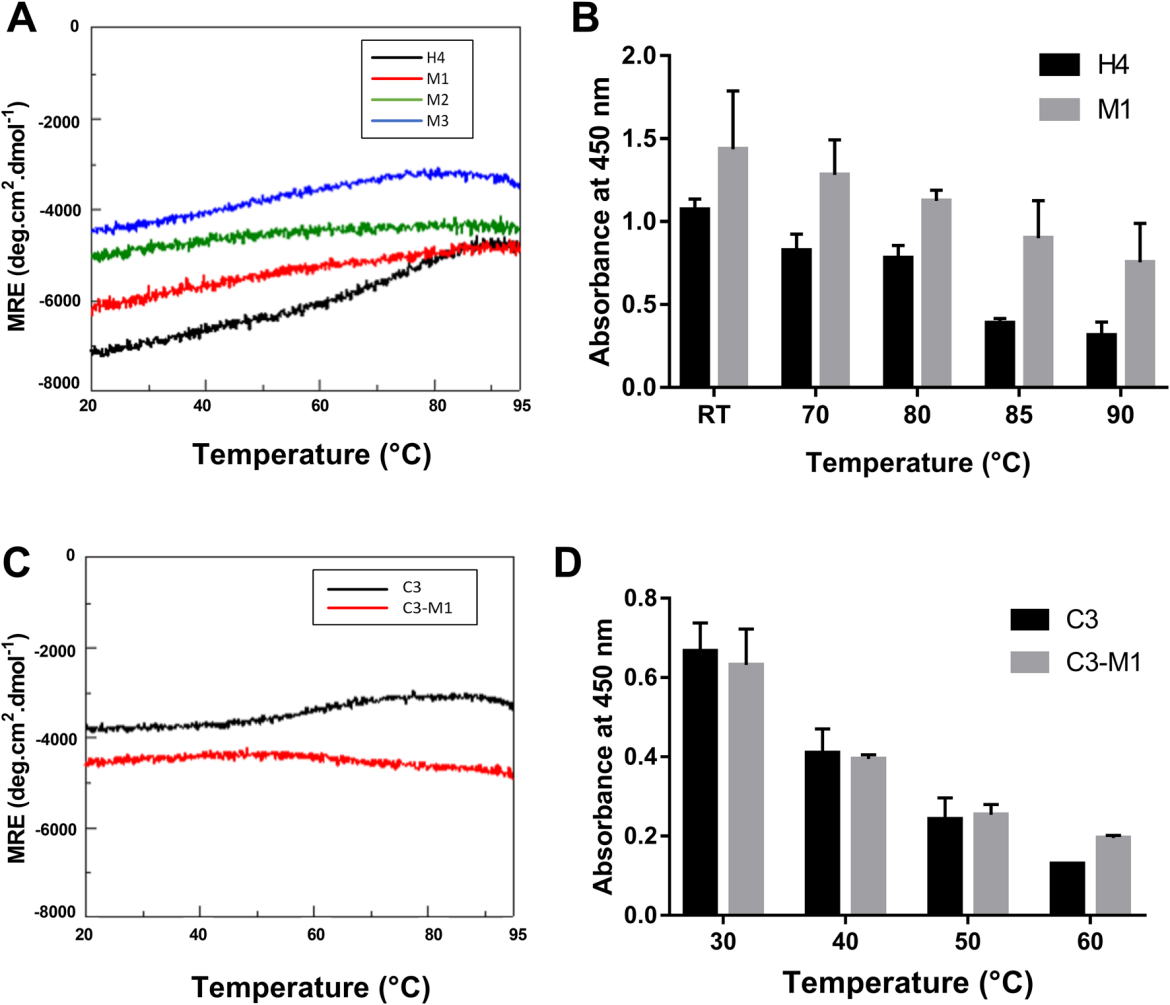
Thermostability of wild-type and mutated Affitins. CD measurements at 222 nm of 0.25 mg/mL wild-type and mutated H4 (**A**) or C3 (**C**) were recorded in 20 mM potassium phosphate buffer at pH 7.4 and at temperatures ranging from 20 to 95 °C. 125 nM H4 or M1 (**B**) or 500 nM C3 or C3-M1 (**D**) were pre-incubated in PBS-T for 1 h at different indicated temperatures before they were incubated for 1 h at room temperature with their specific target. Bindings were detected with anti-RGS-6His-HRP antibody conjugate and absorbance at 450 nm. Data points represent means and error bars represent standard deviation of absorbances achieved from at least three experiments.

The results indicated that wild-type and mutants exhibited high thermostability with similar spectrum profiles (Fig. 7A). Interestingly, we noted that, compared with H4 (in black, Fig. 7A), M1 (in red, Fig. 7A) appeared to display slightly enhanced thermal resistance, as seen by its negligible change in ellipticity at 222 nm from 20 to 95 °C. This increased thermostability of M1 was confirmed using ELISA experiments (Fig. 7B). Increasing the pre-incubation temperature reduced protein binding for both H4 and M1, but the reduction was more pronounced for H4 and the absorbance of M1 was more than 2 times higher than that of H4 after pre-incubation for 1 h at 90 °C (Fig. 7B).

### Effect of mutations at residues 6 and 7 on Affitin properties

Taken together, our results emphasize that the double mutations introduced in M1 not only retain Affitin’s known remarkable properties (pH and thermal stability, strong binding, high production yield) but they also conferred notable improvements. To confirm these observations, we inserted the same mutations into the sequence of another Affitin. To make the following characterizations easier, we used the previously described Affitin C3 that targets human immunoglobulin G (hIgG)^31^. Table 2 gives confirmation that mutating the residues F6 to W6 and L7 to I7 in the sequence of C3 provides the same improvements as those we have already observed with H4: an increase in production yield (3.1-fold) and an increase in the apparent affinity, corresponding to a decrease in the equilibrium association constant K_D_ (6.4-fold). In addition, we also confirmed that C3-M1 retained its excellent stability properties under extreme pH (supplementary Fig. S6) and thermal conditions (Fig. 7C,D). As for H4, and compared with C3 (in black), we observed that C3-M1 (in red) retained high thermostability, as seen by its negligible change in ellipticity at 222 nm from 20 to 95 °C (Fig. 7C). Moreover, for each pre-incubation temperature used from 30 to 60 °C, the absorbance of C3-M1 was higher (at 60 °C) or similar (at 30, 40 and 50 °C) than that of C3 (Fig. 7D). Both C3 and C3-M1 lost their affinity at temperatures of more than 70 °C.

**Table 2:** Characteristics of wild-type and mutated C3.

	Mutations	MM^a^	EC^b^	Yield^c^	K_D_^d^ [Chi2^e^]
C3	/	9068.24	2980	6.5 ± 0.2	615 [0.84]
C3-M1	F6W-L7I	9107.28	8480	20.0 ± 0.3	96.7 [2.56]

^a^Molecular mass (MM) in g mol^−1^.

^b^Molar extinction coefficient (EC) at 280 nm in M^−1^ cm^−1^.

^c^mg of Affitin purified from 1 L of growth medium.

^d^Equilibrium dissociation constant in nM.

^e^Chi2 in RU measured by SPR measurements with concentrations of Affitin from 31.2 to 1000 nM.

## Discussion

Clinical applications can need proteins to exhibit both high affinity and structural stability under harsh conditions. These qualities are even more important if oral administration is considered. Many factors affect the oral bioavailability of a drug, including solubility, absorption, and stability. Gastrointestinal transit is particularly challenging for therapeutic proteins because of the presence of proteases whose mission is to degrade proteins to ensure proper digestion of food and provide tissues and cells throughout the body with essential nutrients.

The goal of this study was first to evaluate the stability of Affitins, a new class of specific affinity binders^12,13^, in simulated gastric (FaSSGF) and intestinal (FaSSIF) fluids. For this purpose, FaSSGF and FaSSIF were prepared based on the updated composition proposed by Biorelevant.com. As proof of concept, we used Affitin H4 which targets hen egg white lysozyme^22,23^. Although rapidly digested in the intestinal medium, H4 appeared to be degraded into stable fragments in gastric medium. In the same simulated conditions, similar stable fragments were obtained with all the other Affitins we tested, while a total disappearance of proteins was observed with the other biological binders we examined, such as a nanobody and an antibody (supplementary Fig. S7). These results further confirm the particularly high stability of Affitins under extreme conditions (e.g. pH 1.6 and the presence of pepsin).

Digestion of Affitins thus generated small fragments that no longer interacted with their target, preventing the oral administration of Affitins during medical processes. Mass spectrometry experiments combined with computer tools allowed us to detect three cleavage sites at different locations in the H4 sequence. Previous work has shown that mass spectrometry can provide in-depth analysis of peptides produced after digestion of proteins (hazelnut allergens)^8^. Subsequently, our purpose was to introduce specific mutations into H4 to optimize the gastric stability of Affitins. Nevertheless, any sequence change may affect the interaction and structure integrity of the protein. It has already been shown that the mutations of F32 (corresponding to F31 in Sso7d) in tyrosine and alanine residues significantly reduced its stability^33^. F32 is a member of the aromatic cluster in the protein core, fundamental for the stability of the native conformation. We considered preferential substitutions of amino acids in proteins that preserve structures and functions^27^ and the probability of two residues being cleaved by pepsin (which particularly affects L and F)^28,29^. The library of Sac7d variants generated to select Affitins is composed of conserved residues and fourteen random residues^23^. It should be noted that only F7 and L31 are randomized residues, thus specifically selected in H4. The other five (F6 and F32, L54, L55 and L58) are conserved in all Affitins. Moreover, as indicated by Correa et al*.*^23^, L31 and F32 did not appear to be directly involved in the interaction with lysozyme but they are part of the binding interface.

Based on this knowledge, the conserved F6 and F32 were replaced by a tryptophan to maintain protein conformation. Being both aromatic and hydrophobic, F and W residues preferred to be buried in hydrophobic protein cores and may be involved in protein stabilities by stacking interactions with other aromatic side chains^27^. In addition, these mutations made it possible to insert an aromatic amino acid known to confer interesting spectroscopic properties^34^. The other residues (F7 and L31, L54, L55 and L58) were substituted by an isoleucine or a valine generally poorly recognized by pepsin^28,29^. Three resulting mutants of H4 (M1, M2 and M3) were successfully expressed using *E. coli* and purified to homogeneity.

Using our simulated gastric fluid FaSSGF containing pepsin, we investigated whether the substitutions introduced protect Affitins against proteolytic digestion. Mutations in M1 (F6W-F7I) did effectively provide resistance to pepsin digestion. On the contrary, we found that the mutations introduced in M2 (L31I-F32W) and M3 (L54I-L55V-L58I) did not cause any resistance against pepsin digestion, although slower degradation was observed for M2. Interestingly, as indicated by mass spectrometry analyses, cleavages in M2 and M3 were not directly at the sites previously identified in the wild-type, but they were very close to them (one or two residues). These results suggest that, if a region of a protein containing proteolytic cleavage sites is particularly exposed, then even the introduction of more stable residues may not be enough to regain stability against the protease. The two sites for pepsin digestion in M2 and M3 could still be highly exposed after both mutations, allowing the enzyme to degrade them.

We further investigated the effect of the three mutations on the structure and function characteristics of H4. We observed that the mutants still adopted a well-defined fold, however the resulting conformations appeared to be different from that of wild-type H4. The difference was slight for M1. Magnitude of spectra may be affected by several factors, including protein concentration and protein purity. But we could not exclude a minor rearrangement of M1, particularly in the β-sheet containing the mutated F6 and F7. On the contrary, a significant shift and decrease in ellipticity intensity were observed for M2 and M3 spectra. These spectrum changes could indicate a decrease in α-helix content, especially for M3 as the mutations were situated in the unique α-helix of H4. They may also be explained by an increase in β-sheet conformation and/or by rearrangement of the β-sheet structure. Huge morphological and spectral diversity in the β-structures has been shown in the far-UV CD region, as β-sheet twisting in a protein greatly contributes to its CD spectrum^35^. As mutations in M1 and M2 are found in the β-sheet parts of H4, they may induce a rearrangement in these structures, explaining the variations in their spectrum, but not necessarily implying a variation in the overall protein structure. In addition, we introduced into the M1 and M2 mutants a Trp amino acid which is known to make significant and distinctive contributions even to the far-UV CD spectrum. Thus, variations in the CD spectrum would not systematically imply variations in the protein’s overall structure neither in protein activity, as seen with M3. However, we found that the substitutions in M2 strongly decreased the binding of the protein to its target. Visual inspection of the three-dimensional structure of the H4-lysozyme complex showed that the residues at these positions are close to the binding interface with its target but they should not interact directly^23^. Even if we performed preferential substitution to conserve structure and function, a slight modification to amino acids (such as L to I and F to W) in this area of interaction seemed to impact binding affinity. M2 mutations did not drastically modify the pattern of the CD spectra, suggesting that they did not destabilize the protein. In addition, the M2 spectrum showed similar shape and magnitude variations compared to the M3 spectrum, while the affinity of M3 was not affected by the corresponding mutations. Thus, either the mutations in M2 induce minor changes in the protein conformation which interfere with its target binding, or the corresponding amino acids (L31 and/or F32) are actually involved in the target interaction. Whatever the reason, we cannot consider introducing these mutations into Affitins, as not only do they not provide proteolysis resistance (in M2 and M3), but they also lead to a decrease in protein affinity (in M2).

Interestingly, the substitutions performed in M1 made it possible to trigger valuable properties, thus giving rise to several improvements. First, the mutations provided resistance to pepsin digestion. Second, the association efficiency of M1 was 2 times greater than that of the wild-type protein. Third, the production yield of M1 increased more than 4 times compared to H4. Finally, we showed that M1 retained the significant stability of H4 under extreme pH and temperature conditions. We used homology modelling, using the crystal structure of Affitin H4 with lysozyme (PDB ID: 4CJ2)^23^ and PyMOL software to visualize the impact of the mutations (Fig. 2C). Replacing F6 and F7 (in red) by a W and a I (in yellow) respectively did not seem to induce major conformational changes. For instance, although the side chain of F6 was oriented toward the inside of the H4 structure, its mutation into a W seemed surprisingly well suited to the minimal steric hindrance from neighbor residues (K13 and W8). Interestingly, this substitution may induce minor conformational changes that slightly stabilize the 3D structure. Furthermore, W6 may induce a displacement of W8, which is in close proximity with the lysozyme in the complex, less than 3 Å, thereby reinforcing the affinity of the protein for its target. This effect may therefore be similar to what has been observed in antibodies with the so-called “second-sphere residues” which have an influence on binding sites while being outside^36^. The hydrolysis inhibition induced by mutations in M1 would probably be mainly due to replacing amino acids preferentially cleaved by pepsin (like F6 and F7) with amino acids poorly recognized by the enzyme^28,29^. But we cannot exclude that the minor conformational changes, as discussed above, also protect the protein from pepsin cleavage at this position by increasing stability.

To validate that the remarkable effects of residue substitutions in M1 can be transposed to another Affitin, we inserted the same mutations in the Affitin C3^31^. In addition to a noticeable production yield increase, we found that C3-M1 has a strong increase in its apparent affinity. CD experiments showed that C3-M1 displays a different spectrum profile to C3, while maintaining its target affinity and high stabilities towards pH and temperature variations. Proteolysis resistance was observed for C3-M1 between residues 6 and 7. Nevertheless, the fourteen random residues making up the different Affitins lead to the presence of a L at position 8 in C3 sequence, which was cleaved by pepsin.

Previous work has already shown that the randomization of about 20% of the Sac7d-derived sequence does not alter the overall fold of the protein and that several tolerated mutations could be considered^31^. Our results showed that all mutant proteins are hyperthermostable in phosphate buffer and are highly resistant from low to high pH, in good agreement with the Sul7d protein family^31,37,38^. Altogether, our findings highlight that substituting F6 and residue 7 in Affitins with respectively W and I produced a mutant M1 that acquired outstanding properties. As these residues are not on the central surface of interaction with the target, we can consider systematically inserting them into the libraries, issued from Sul7d proteins, thereby programming these improvements in all selected Affitins. Moreover, to date, no W is present in the sequence, thus inserting a W residue would have additional advantages, such as higher absorbance at 280 nm and exploitable fluorescence properties^34^. Rational protein engineering has already contributed to generating mutations with enhanced properties^5,39–41^. Frequently, this genetic approach can also simultaneously elucidate the structure–function relationship of a protein^42–44^, thus providing better guidance for future design proposals. Niu et al*.* found that substituting three residues (L99, L162 and E230) improved pepsin resistance and that in addition, some of them increased their catalytic efficiency 1.3–2.4-fold and improved their stability at 60 °C and pH 1–2^39^. Camilloni et al*.* showed that the propensity for aggregation of a protein can be modulated by mutating specific residues that change the average protection of its aggregation-prone surface residues without affecting its structure and stability^42^. Ashraf et al*.*^43^ and Han et al*.*^40,44^ demonstrated that modifying non-catalytic residues can promote favourable catalytic behaviour and that substituting these residues can provide prospective candidates for industry applications.

## Conclusion

The oral delivery of therapeutic protein drugs is highly challenging as the digestive tract does not differentiate between therapeutic and non-therapeutic proteins. As only proteins that persist in the gastrointestinal transit with an intact conformation can be considered for the oral route, simulated digestion assays are frequently used to discriminate between drugs. In particular, biorelevant media have quickly emerged as a reliable, rapid and relatively inexpensive tool for supporting the drug discovery and development process. Furthermore, by mimicking physiological conditions, these in vitro models are widely recommended and represent a satisfying answer to the increasing societal pressure for the rational use of animals for research^6,8,24,45^. Our study highlighted that biorelevant in vitro simulated media and in silico modelling made it possible to investigate the gastrointestinal degradation of therapeutic proteins with the aim to predict their oral behaviour, constituting a first step in understanding their in vivo pharmacokinetics. We showed how difficult the oral administration of Affitins can be without further improvements, as for most proteins, due to their low resistance to gastric and intestinal proteases. It could be more appropriate to consider encapsulation or to use another route of administration, such as pulmonary or sublingual pathways, but also the more commonly used parenteral protein delivery.

Nevertheless, we have successfully identified beneficial mutations for proteolytic resistance that also lead to additional improved properties, such as affinity, stability and production level. These improvements are particularly sought after in the development of targeting agents for diagnosis and therapy, such as Affitins.

## Materials and methods

### Materials

Unless otherwise specified, the chemicals were purchased from Sigma-Aldrich, and the enzymes and buffers for molecular biology were purchased from Thermo Fisher Scientific. Oligonucleotides were purchased from Eurofins.

### Protein mutagenesis

Reactions were carried out using the Phusion site-directed mutagenesis protocol, as described by the manufacturer (Thermo Scientific), in a final reaction volume of 50 µL with 10 ng of plasmid DNA derived from pQE30 (Qiagen) and encoding Affitins (pFP1001-H4^23^ or pFP1001-C3^31^). Final reaction mixtures consisted of 1 × concentrated Phusion HF buffer, 0.2 mM dNTP-Mix, 4% DMSO, 0.25 µM forward primer and reverse primer (stock solutions dissolved in ddH_2_0) and 1 unit of Phusion Hot Start II DNA polymerase. The primers used are described in Table S1. PCR reactions were carried out under the following conditions: a) initial denaturation: 98 °C (30 s); b) cycling (25 passes in total): 98 °C (30 s), 60 °C (50 s) and 72 °C (150 s) c) final elongation: 72 °C (10 min). Following PCR, the samples were analysed using agarose gel electrophoresis (100 V; 1 h) (1% (w/v) agarose in TAE buffer, using a 1 kb DNA ladder as standard for size determination. After confirmation of the correct band sizes, PCR products were digested for 1 h at 37 °C by 1 µL DpnI (100 U/µL). The PCR product was then transformed into the *E. coli* DH5αLacIq strain (Invitrogen) and some colonies were amplified using a Wizard Plus SV MiniPrep DNA purification system Kit (Promega). Concentrations of purified PCR products were determined by absorbance measurements at 260 nm using a NanoDrop spectrophotometer. DNA samples were sent for sequencing (Eurofins) to verify the amino acid sequences.

### Protein production and purification

Protein preparations were performed similarly for mutated and original wild-type proteins as described previously^14^ with modifications. Competent *E. coli* DH5αLacIq were transformed by heat shock at 42 °C with the plasmid coding for the protein and grown overnight in stirring preculture (50 mL in 2YT medium containing 100 µg/mL ampicillin, 25 µg/mL kanamycin and 1% glucose at 37 °C). 20 mL of preculture were then inoculated into 1 L of 2YT medium (with 100 µg/mL ampicillin, 25 µg/mL kanamycin, 0.1% glucose). Expression was induced when the absorbance (OD_600_) reached 0.8- 1.0 by 0.5 mM IPTG, and cultures were shaken at 30 °C for 3 h. Cultures were harvested by centrifugation and the bacterial pellets were resuspended in lysis buffer (Tris–HCl 20 mM pH 7.4, 500 mM NaCl, 25 mM imidazole) and disrupted by several gentle sonications. Cell debris was pelleted by centrifugation, and supernatants, containing the protein of interest, were incubated with 1.5 mL of nickel affinity resin (Ni–NTA, GE Healthcare) at room temperature for 1 h. Proteins containing six N-terminal histidine residues were purified on a 5 mL HiTrap column. Elutions were performed with 5 mL of PBS (137 mM NaCl, 2.7 mM KCl, 8 mM Na_2_HPO_4_, 2 mM KH_2_PO_4_, pH 7.4) with 250 mM Imidazole. Proteins were further purified by size-exclusion chromatography on a Superdex 75 column (GE Healthcare) equilibrated with PBS. Fractions were pooled and concentrations were determined using a NanoDrop spectrophotometer and the corresponding molar extinction coefficient for each Affitin (Tables 1 and 2). Proteins were used for biochemical characterization.

### Simulated gastric fluid digestion assay

Gastric digestion media corresponding to the fasted state were prepared according to the instructions provided by Biorelevant.com Ltd and consisted of a solution made by dissolving 0.06 mg/mL of FaSSIF, FeSSIF and FaSSGF powder, purchased from Biorelevant.com, in 34.2 mM NaCl at a pH of 1.6 adjusted with HCl. Then pepsin was added extemporaneously (from 0.025 to 3.2 mg/mL). Affitins were added at a final concentration of 0.5 mg/mL and were then incubated for 30 min at 37 °C with stirring. Reactions were quenched with 200 mM Na_2_CO_3_. Samples were stored at − 20 °C until use.

### Simulated intestinal fluid digestion assay

Intestinal digestion media corresponding to the fasted state were prepared according to the instructions provided by Biorelevant.com Ltd and consisted of a solution made by dissolving 2.24 mg/mL of FaSSIF, FeSSIF & FaSSGF powder in a buffer containing NaOH 10 mM, NaH_2_PO_4_ 28.66 mM and NaCl 105.88 mM at pH 6.5. Then pancreatin was added extemporaneously (from 0.01 to 10 mg/mL). Affitins were added at a final concentration of 0.5 mg/ml and were then incubated for 30 min at 37 °C with stirring. Reactions were quenched by heating at 100 °C for 10 min. Samples were stored at -20 °C until use.

### Mass spectrometry analysis

Affitins were prepared at a concentration of 0.5 mg/mL with or without pepsin digestion. An SDS-PAGE electrophoresis gel with 15% acrylamide was made to ensure that the proteins had been well digested with pepsin. 100 μL of sample were put in restrictors themselves placed in LC vials (Macherey Nagels). LC–MS/MS analyses were performed as described previously with minor modifications^46^, on a Synapt G2 HRMS Q-TOF mass spectrometer equipped with an electrospray ionization (ESI) interface operating in positive mode, and an Acquity H-Class UPLC device (Waters Corporation, Milford, MA, USA). Peptides were separated on a BEH C_18_ column (2.1 × 100 mm; 1.7 µm; Waters Corporation) with a linear gradient of mobile phase B (100% acetonitrile) in mobile phase A (5% acetonitrile), each containing 0.1% formic acid, and at a flow rate of 400 µL/min. Mobile phase B was kept constant at 1% for 1 min, then linearly increased from 1 to 99% for 10 min, kept constant at 99% for 1 min, returned to the initial condition for 1 min, and kept constant for 2 min before the next injection. The column temperature was maintained at 60 °C throughout the run. Peptides were then detected by the mass spectrometer with the ESI interface operating in the positive ion mode (capillary voltage, 3 kV; desolvatation gas (N_2_) flow and temperature, 900 L/h and 400 °C; source temperature, 150 °C). Data were acquired at the rate of 4 spectra/sec and the acquisition window was set from m/z 50 to 4000. For MS/MS experiments, major precursor ions were selected according to their specific m/z and then fragmented with a collision energy set at 30 V. The multiple charge state distributions of intact proteins were deconvoluted by using the MaxEnt1 extension of the MassLynx software (version 4.1, Waters Corporation) and generated MS/MS spectra were manually analysed to find the sequence coverage of each protein^47^.

### Polyacrylamide gel electrophoresis

The impact of pepsin digestion was studied by Sodium Dodecyl Sulfate—polyacrylamide gel electrophoresis (SDS-PAGE) either by 15% acrylamide gels in Tris/glycine/SDS running buffer (glycine–SDS–PAGE) or by 10% acrylamide gels in Tris/tricine/SDS running buffer (tricine-SDS-PAGE). Samples (10 µL, 5 µg protein) were mixed with 4× loading buffer with β-mercaptoethanol and heated for 10 min at 100 °C. Gels were run in a Mini-PROTEAN Tetra cell system (Bio-Rad) for 1 h at 150 V (glycine-SDS-PAGE) or for 1 h 30 at 120 V (tricine-SDS-PAGE). All gels were stained overnight using PageBlue Protein Staining Solution (Thermo Fisher Scientific) and destained with different water baths. Image analyses of gels were performed using ImageLab software (version 6.0.1, Bio-Rad). Band sizes were determined by Precision Plus Protein all blue markers from Bio-Rad.

### Target affinity and thermostability by Enzyme-linked immunosorbent assay (ELISA)

To evaluate the binding specificity of mutated Affitins against its targets and their thermostability, ELISA experiments were carried out as described previously^30^ with modifications. A Maxisorp plate (Nunc) was coated for 1 h with 100 μL of chicken lysozyme (Sigma) or hIgG (Tegeline, kindly provided by Bernard Vanhove, Nantes University) at 1 mg/mL and washed three times with PBS pH 7.4 before an overnight saturation with BSA 0.5%. To assess binding specificity, 100 μL of Affitins with concentrations ranging from 3.9 nM to 1 µM in PBS pH 7.4 containing 0.1% Tween 20 (PBS-T) were added to the corresponding well and incubated for 1 h at room temperature. For thermostability evaluation, Affitins (125 nM) were first incubated in PBS-T for 1 h at different temperatures (from room temperature to 90 °C) and immediately cooled down on ice, then 100 µL were added to the corresponding well and incubated for 1 h at room temperature. In both experiments, Affitins were tested for non‐specific binding against BSA without target. After washing with PBS-T, the plates were incubated for 1 h at room temperature with 100 μL anti‐RGS‐6His antibody (Qiagen, dilution 1:5000), conjugated with horseradish peroxidase (HRP). After washing with PBS-T, bound proteins were detected using 100 µL of *ortho*‐phenylenediamine (OPD) solution (1 mg/mL OPD, 0.05% H_2_O_2_, 100 mM sodium citrate pH 5.0) as the substrate. Absorbance at 450 nm was recorded with a plate reader (Tecan infinite M200 Pro).

### Target affinity by Surface Plasmon Resonance (SPR)

Association and dissociation of wild-type and mutated Affitins on their target were conducted at 25 °C using SPR experiments with a BIAcore T200 instrument. Targets (lysozyme or hIgG) were immobilized on a CM5 chip by amine coupling on two independent channels. Briefly, an NHS / EDC mix was injected on to a CM5 chip to generate reactive ester groups. Targets were diluted to 20 μg/mL in sodium acetate buffer pH 5.5 or sodium acetate buffer pH 4.5 respectively and injected on to the activated chip on channels 2 and 4. Lysozyme and hIgG were immobilized at 200 RU and 1200 RU respectively. The residual ester groups were inactivated by injecting a 1 M ethanolamine solution pH 8.5 for 10 min. The running buffer was HBSEP pH 7.4 (10 mM Hepes, 150 mM NaCl, 2 mM EDTA and 0.05% P20). Affitins (H4, M1, M2 and M3) were injected in dose response on to lysozyme at the following concentrations: 500, 250, 125, 62.5, 31.2, 15.6 nM. Associations were measured for 2 min and dissociations for 7 min. The flow rate was set at 30 μL/min and the regenerations were carried out by injection for 30 s of a 10 mM NaOH solution. Affitins C3 and C3-M1 were injected in dose response on to hIgG at the following concentrations: 1000, 500, 250, 125, 62.5, 31.2 nM. Associations were measured for 2 min and dissociations for 5 min. The flow rate was set at 30 μL/min and the regeneration carried out by injecting for 30 s a solution of 1 M ethanolamine pH 8.5. The resulting data were evaluated with BIAevaluation 3.1 and the kinetic parameters k_on_, k_off_, and KD were calculated.

### Structural analysis by circular dichroism (CD)

Far-UV CD spectroscopy was used to both determine the secondary structures of WT and mutated Affitins and evaluate their stability at different pH and temperatures. Measurements were carried out in a Jasco J‐810 instrument (Jasco, Lisses, France), using a quartz cell with a path length of 0.2 cm (Hellma, Paris, France). For pH stability, 0.25 mg/mL of Affitins were incubated overnight at room temperature in 20 mM sodium phosphate buffer at pH ranging from 4 to 10 or in HCl 1 N for pH 0. CD spectra were performed at 20 °C. For data processing, three consecutive scans were collected for each measurement from 210 to 250 nm, and average spectra were smoothed and stored. Data were collected as observed ellipticity in millidegrees (mdeg) or mean residue ellipticity (MRE) in deg cm^2^ dmol^−1^ versus wavelength (nm). Thermal unfolding was monitored at 222 nm in potassium phosphate buffer, pH 7.4 in a temperature measurement mode starting from 20 to 95 °C with a scanning rate of 1 °C/min. Data were collected as MRE in deg cm^2^ dmol^−1^ versus temperature (°C). MRE was calculated using the following equation: MRE = (observed ellipticity (mdeg) x MM) / (10 × d x c x N) where MM is the molecular mass for each Affitin (Tables 1 and 2), d is the path length in cm, c is the protein concentration in mg/mL and N is the number of residue (79).

## Supplementary information

Supplementary Information
