# Supplementary material for: Characterization of Affitin proteolytic digestion in biorelevant media and improvement of their stabilities via protein engineering
Source: Sci Rep. 2020 Nov 12;10:19703. doi: 10.1038/s41598-020-76855-z (PMC7661517; doi:10.1038/s41598-020-76855-z)
Supplement: Supplementary file 1 — Supplementary Information [file 41598_2020_76855_MOESM1_ESM.pdf]

Supplementary Information

Characterization of Affitin proteolytic digestion in biorelevant media and improvement of their stabilities via protein engineering

Aurélie Loussouarn[1], Ghislaine Béhar[1], Frédéric Pecorari[1], Mikael Croyal[2,3], Axelle Renodon-Cornière[1]

[1] CRCINA, INSERM, CNRS, Université d'Angers, Université de Nantes, Nantes, France. [2] NUN, INRA, CHU Nantes, UMR 1280, PhAN, IMAD, CRNH-O, F-44000 Nantes, France. [3] CRNH-O Mass Spectrometry Core Facility, F-44000 Nantes, France.

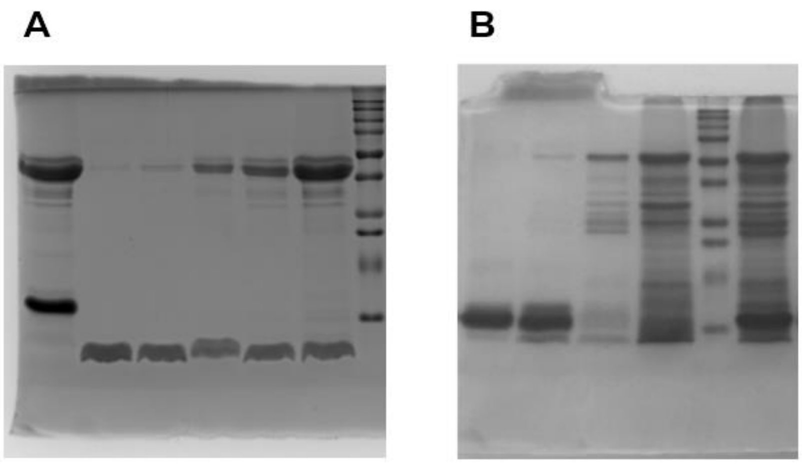

Supplementary Figure S1. Full-length gel images shown in Fig. 1. Full-length gel images of SDS-PAGE performed under reducing conditions and stained with Coomassie brilliant blue.

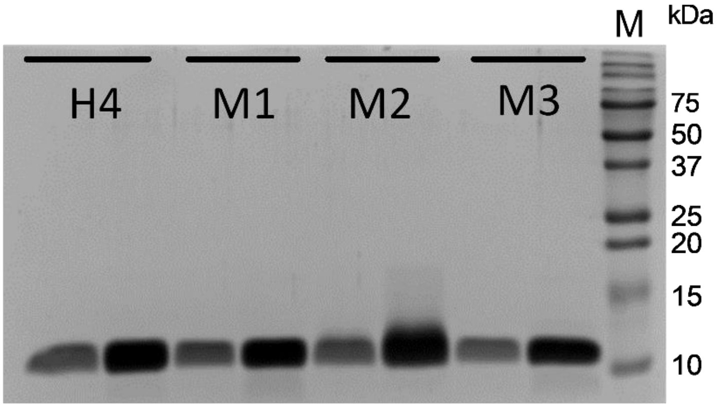

Supplementary Figure S2. Purities of wild-type and mutated H4 by SDS-PAGE. Purified proteins (1 and 3 µg) were separated by SDS−PAGE (15 % acrylamide gel) under reducing conditions and stained with Coomassie brilliant blue. M: Molecular mass markers.

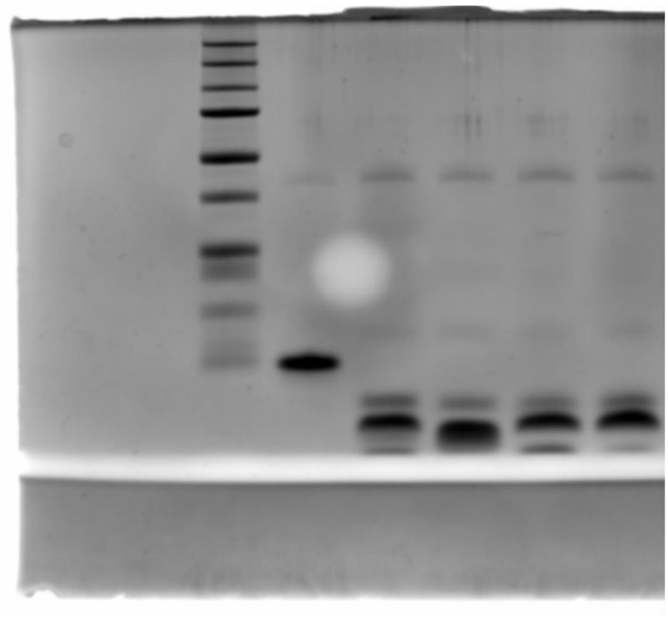

Supplementary Figure S3. Full-length gel image shown in Fig. 4A. Full-length gel image of SDS-PAGE performed under reducing conditions and stained with Coomassie brilliant blue.

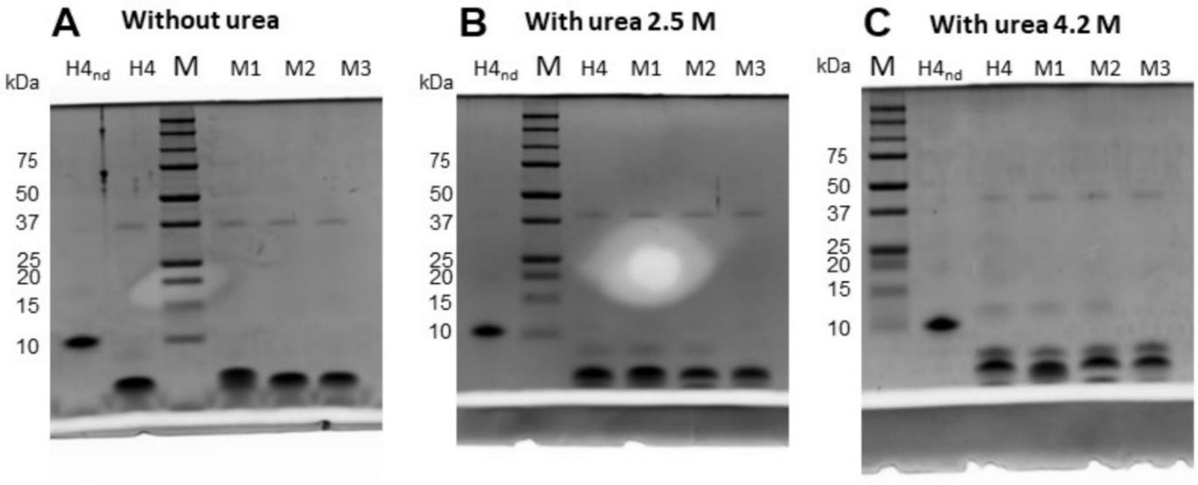

Supplementary Figure S4. Full-length gel images of Tricine SDS-PAGE in absence (A) or in presence of 2.5 M urea (B) or 4.2 M urea (C) under reducing conditions and stained with Coomassie brilliant blue.

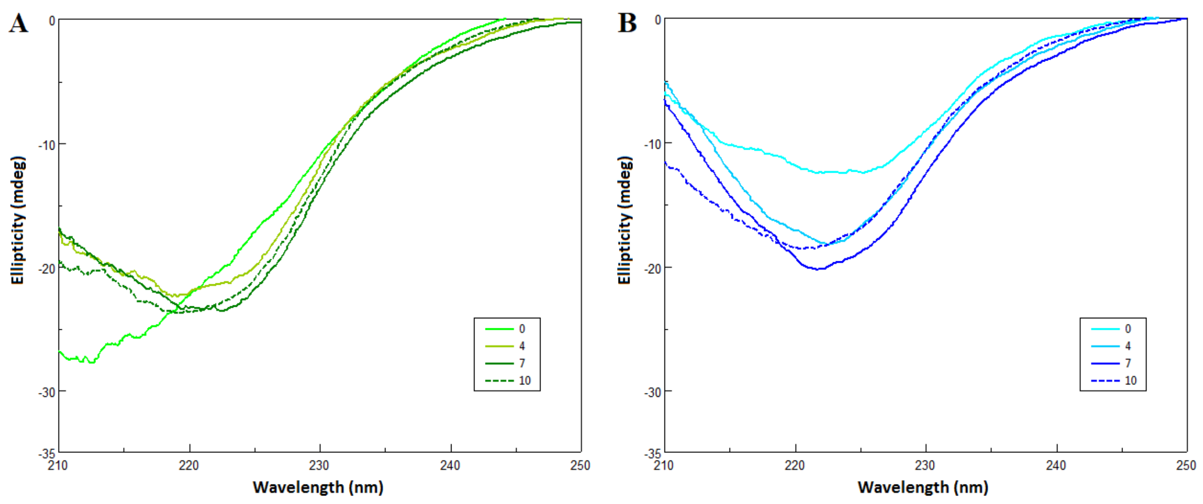

Supplementary Figure S5. Secondary structure of M2 and M3 mutants by circular dichroism.

Far-UV CD spectra of 0.25 mg/mL of M2 (A) and M3 (B) were recorded at 20 °C in 20 mM sodium phosphate buffer at different pH ranging from 0 to 10.

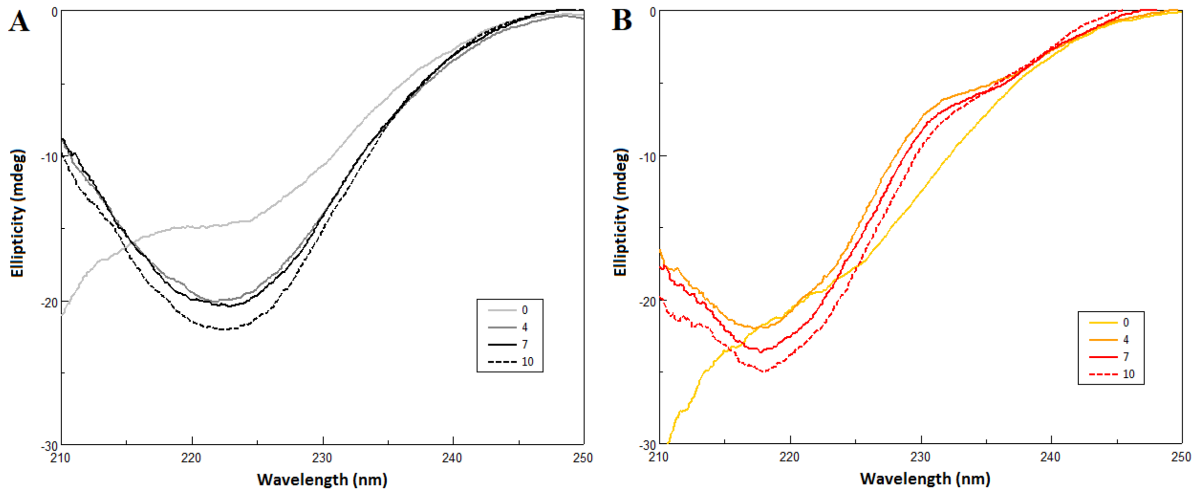

Supplementary Figure S6. Secondary structure of wild-type and mutated C3 by circular dichroism. Far-UV CD spectra of 0.25 mg/mL of C3 (A) and C3-M1 (B) were recorded at 20 °C in 20 mM sodium phosphate buffer at different pH ranging from 0 to 10.

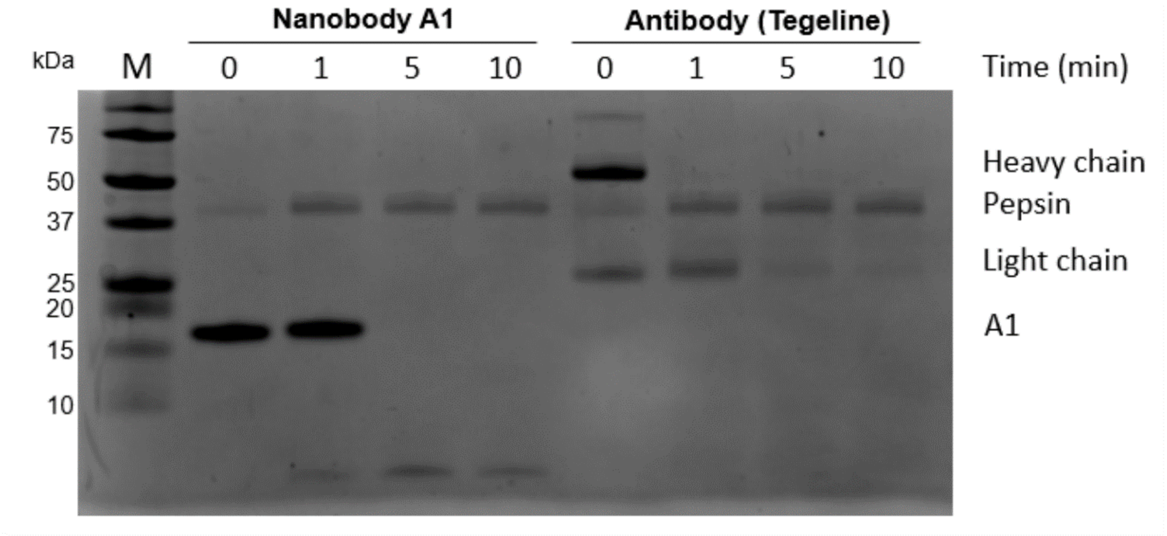

Supplementary Figure S7. Proteolytic digestion of a nanobody and an antibody by Pepsin in FaSSGF. Proteins (0.25 mg/mL, 2.5 µg) were separated by SDS–PAGE under reducing conditions and stained with Coomassie brilliant blue after they were incubated at 37 °C with 0.1 mg/mL pepsin in FaSSGF medium (pH 1.6) for different time periods (0, 1, 5 and 10 min). Lane 1: Molecular mass markers (M); From lane 2 to lane 5: Nanobody A1 (described in Prantner, A.M. *et al.* Anti-Mesothelin Nanobodies for Both Conventional and Nanoparticle-Based Biomedical Applications. *J. Biomed. Nanotechnol.* **11**, 1201-1212 (2015)) is fully digested within 5 min. From lane 6 to lane 9: we used Tegeline (the target of C3 Affitin) as antibody which was also totally digested within 5 min.

Supplementary Table S1 – Amino acid sequences of Affitins with wild-type or mutated residues in bold.

Affitins	Amino acid sequences
H4	MRGSHHHHHHGSVKVK**FF**WNGEEKEVDTSKIVWVKRAGKSV**LF**IYDDNGKNGYGDVTEKDAPK E**LL**DM**L**ARAEREKKLN
M1	MRGSHHHHHHGSVKVK**WI**WNGEEKEVDTSKIVWVKRAGKSVLFIYDDNGKNGYGDVTEKDAPK ELLDMLARAEREKKLN
M2	MRGSHHHHHHGSVKVKFFWNGEEKEVDTSKIVWVKRAGKSV**IW**IYDDNGKNGYGDVTEKDAPK ELLDMLARAEREKKLN
M3	MRGSHHHHHHGSVKVKFFWNGEEKEVDTSKIVWVKRAGKSVLFIYDDNGKNGYGDVTEKDAPK E**IV**DM**I**ARAEREKKLN
C3	MRGSHHHHHHGSVKVK**FL**LNGEEKEVDTSKIRDVCRQGKNVKFLYNDNGKYGAGNVDEKDAPK ELLDMLARAEREKKLN
C3-M1	MRGSHHHHHHGSVKVK**WI**LNGEEKEVDTSKIRDVCRQGKNVKFLYNDNGKYGAGNVDEKDAPK ELLDMLARAEREKKLN

Supplementary Table S2 – Primer sequences used for site-directed mutagenesis of H4.

Mutants	Mutations		nucleotide sequence (5'-3')
M1	F6W-F7I	F	CGGATCCGTCAAGGTGAAAT**GGA**TCTGGAACGGCGAAGAAAAAGAAGTGG
		R	CCACTTCTTTTTCTTCGCCGTTCCAGATCCATTTCACCTTGACGGATCCG
M2	L31I-F32W	F	GCGTGCGGGCAAAAGTGTG**A**TCT**GG**ATCTACGACGACAACGGC
		R	GCCGTTGTCGTCGTAGATCCAGATCACACTTTTGCCCGCACGC
M3	L54I-L55V-L58I	F	CCGAGAAAGATGCCCCGAAAGAG**A**T**TG**TAGATATG**A**T**T**GCGCGTGCGGAACGCG
		R	CGCGTTCCGCACGCGCAATCATATCTACAATCTCTTTCGGGGCATCTTTCTCGG

The bold nucleotides correspond to the substituted bases
